# Cell Therapy for Retinal Dystrophies: From Cell Suspension Formulation to Complex Retinal Tissue Bioengineering

**DOI:** 10.1155/2019/4568979

**Published:** 2019-01-23

**Authors:** Karim Ben M'Barek, Christelle Monville

**Affiliations:** ^1^INSERM U861, I-Stem, AFM, Institute for Stem Cell Therapy and Exploration of Monogenic Diseases, 91100 Corbeil-Essonnes, France; ^2^UEVE U861, I-Stem, AFM, Institute for Stem Cell Therapy and Exploration of Monogenic Diseases, 91100 Corbeil-Essonnes, France; ^3^CECS, I-Stem, AFM, Institute for Stem Cell Therapy and Exploration of Monogenic Diseases, 91100 Corbeil-Essonnes, France

## Abstract

Retinal degeneration is an irreversible phenomenon caused by various disease conditions including age-related macular degeneration (AMD) and retinitis pigmentosa (RP). During the course of these diseases, photoreceptors (PRs) are susceptible to degeneration due to their malfunctions or to a primary dysfunction of the retinal pigment epithelium (RPE). Once lost, these cells could not be endogenously regenerated in humans, and cell therapy to replace the lost cells is one of the promising strategies to recover vision. Depending on the nature of the primary defect and the stage of the disease, RPE cells, PRs, or both might be transplanted to achieve therapeutic effects. We describe in this review the current knowledge and recent progress to develop such approaches. The different cell sources proposed for cell therapy including human pluripotent stem cells are presented with their advantages and limits. Another critical aspect described herein is the pharmaceutical formulation of the end product to be delivered into the eye of patients. Finally, we also outline the future research directions in order to develop a complex multilayered retinal tissue for end-stage patients.

## 1. Introduction

Lining the back of the eye, the retina is a light-sensitive tissue composed of several neuronal layers that convert light stimuli into electrical impulses that are further processed and integrated. The resulting signal is then transmitted to the brain through the optical nerve. Photoreceptors (PRs), which convert these light inputs, are in contact with a specific epithelial layer, the retinal pigment epithelium or RPE, which provides a trophic support and maintains PR homeostasis. Among other functions, the RPE is involved in the elimination of photoreceptor debris, the secretion of growth factors, the transport of nutrients, and the recycling of proteins involved in the visual cycle [1, 2]. A number of defects altering the functions of this RPE layer lead to some forms of PR degeneration. The loss of PRs, due to their malfunctions or to a primary dysfunction or death of RPE cells, might impact the vision of affected patients and in some cases ultimately lead to blindness. Age-related macular degeneration (AMD) and retinitis pigmentosa (RP) are the main conditions in which PRs degenerate. Depending on the stage of the disease, the replacement of the RPE layer and/or the PRs through cell therapy is a promising therapeutic alternative [3]. This review describes the current research and recent development of such treatments.

## 2. Retinopathies

### 2.1. Retinitis Pigmentosa

RP is a heterogeneous group of inherited disorders that could affect either the RPE or the PRs or both [4–6]. To date, more than 60 genes have been involved in RP (https://sph.uth.edu/retnet/disease.htm). Taken individually, each monogenic dystrophy is rare but the global prevalence for RP is comprised between 1/3500 and 1/4000 [7, 8]. Mutations affecting RPE functions account for 5% of all RP [3]. Though the clinical picture is variable according to the nature of the mutation, patients usually experience night vision loss followed by the reduction of visual field from the periphery to the centre (named tunnel vision). At late stages, central vision might also be lost leading to blindness [7–9]. Genes involved in RP could affect essential processes like the phototransduction cascade, the visual cycle, and the recycling of PR debris, which engenders an impairment of the whole pathway and the accumulation of intermediates. Genes involved in RP might also alter the structure of the cells like the connecting cilium [9]. In the US and Europe, regulatory agencies approved the first gene therapy to treat RPE65-mutated patients [10]. However, this treatment is susceptible to treating only a minority of patients.

### 2.2. Age-Related Macular Degeneration

AMD is the other condition in which PRs degenerate. It represents the leading cause of blindness in Western countries. The elderly population is at risk with 12% of people older than 80 years being affected. As the life expectancy increases worldwide, AMD is becoming a global burden [11]. Current projections estimate that the number of patients with AMD will grow to 196 million in 2020 and could reach 288 million in 2040 [11]. The aetiology of AMD is multifactorial with a combination of genetic and environmental causes. A family history of AMD is the second largest risk factor after age. Environmental causes include hypertension, obesity, diet, sunlight exposure, chronic inflammation, and smoking [12, 13]. The disease usually begins in one eye but becomes quickly bilateral in 80% of patients [14]. RPE cells appear to be altered in AMD as well as Bruch's membrane localized at the basal side of the RPE. Accumulation of deposits close to RPE cells, inflammatory modulation, and oxidative damage seem to be at the origin of RPE alterations [15]. AMD could be classified into 2 types: the wet and dry forms. The dry form concerns the vast majority of patients (90%) and could evolve at late stages to geographic atrophy (GA) [16, 17]. GA is characterized by areas where RPE cells are lost, leading to degeneration of PRs. The wet form involves the formation of abnormal blood vessels, is susceptible to leakage, and damages Bruch's membrane, PRs, and RPE cells [18, 19]. These abnormal vessels could also cause hemorrhages [20]. Damage mainly occurs in PRs located in the macular area, thus affecting the central vision. There is no curative treatment for the dry form of AMD. Regarding the wet form, surgical intervention could be done to remove the abnormal vessels but recurrences could appear after a while. Anti-VEGF treatments could also slow down the disease progression and thus preserve the vision but are expensive and need to be administered frequently [20]. However, dead PRs will not be restored by such an approach.

For the abovementioned diseases, a specific cell therapy strategy could be applied depending on the disease stage. For early cases of AMD, where RPE cells are lost and Bruch's membrane is damaged but PRs are still preserved, an RPE cell therapy could be applied. When PRs are also lost, a combination of PR and RPE cell therapies corresponds to the more appropriate treatment. For RP associated with RPE defects, RPE cell therapy should be applied when PRs are still preserved. Otherwise, a complete therapy with RPE and PRs has to be applied, as for other forms of RP.

## 3. Cell Sources

**Figure 1:**
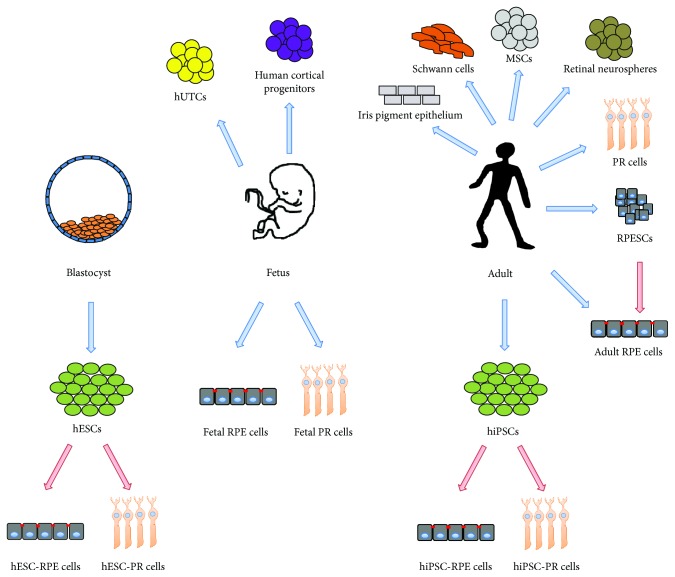
Scheme recapitulating the different cell sources that could be used for the cell therapy of the eye.

The first demonstration of an RPE cell therapy was performed in monkeys in the early 80s [21, 22] and confirmed in rats [23]. Since then, different sources for cell therapy were proposed including adult and fetal, as well as pluripotent stem cells (Figure 1). A large part of the efforts has been directed toward the development of a consistent source for RPE cell therapy, as these cells could be used as a proof of concept for retinal cell therapy.

### 3.1. Fetal Cell Sources

Human fetal RPE cells could be harvested from fetuses (10- to 21-week gestational age). These cells could be then passaged up to 10 times [24–26]. Such fetal RPE cells were transplanted in the Royal College of Surgeons (RCS) rat [27]. This rat carries a mutation in the *MERTK* gene coding for the transmembrane receptor tyrosine kinase MERTK [28], which engenders a functional defect in RPE cells (lack of elimination of PR debris) [29] and PR cell death. Human fetal RPE cells (from 10- to 16-week gestational fetuses) rescued the phenotype of RCS rats when transplanted as a cell suspension into the subretinal space (between RPE and PR layers) [27]. Indeed, histological examination revealed areas of preserved PRs that were surrounding the grafting site. Human fetal RPE cells, when transplanted into the retina, become functional and are able to eliminate the PR debris [30]. Fetal allogeneic RPE were grafted in AMD patients without any visual improvements [31, 32]. However, immune rejection might explain this lack of improvement or the advanced stage of degeneration [33].

Fetal allogeneic sheets containing both RPE and the neural retina were grafted in patients with RP and AMD [34, 35]. No improvement was observed in a first study, and in a second one, 7 out of 10 patients showed improvements in the ETDRS scale (early treatment of diabetic retinopathy study scale, a standardized measure of visual acuity) but not when using multifocal electroretinography (ERG) [35]. The patients were not immunosuppressed, and a decrease in pigmentation with a progressive loss was reported suggesting concerns with the graft survival [35].

Procurement of RPE cells or PRs obtained from human fetuses is regulated and needs ethical agreement from the civil society. This agreement is extremely variable from one country to another making its use, as a cell source, difficult. Moreover, the Declaration of Helsinki has set the requirements for use of human materials: informed consent; no incentive for abortion; procurement of a human material that, if not used for research, would be discarded; review of the protocol from ethical committees; a detailed medical history of the donor; and a complete separation of the donor and the recipient [36]. There is also a variability due to the stage of development of the fetus from which RPE or PRs are obtained. All these constraints lead to the abandonment of this source.

### 3.2. Adult Cell Sources

Human adult RPE cells could be obtained directly from cadavers (allogeneic RPE) or from patients (autologous RPE). The ARPE-19 cell line, commonly used in research laboratories, has been obtained from a 19-year-old man who died following a road accident (in 1986). Following transplantation into the RCS rat, PRs were maintained compared to the nontreated control and the electrical response to light of the retina remained preserved [37, 38]. Besides this cell line, adult RPE cells could be obtained from cadavers [13]. This kind of adult RPE cells is functional in RCS rats [39]. The age of the patient who donated his cadaver was investigated to evaluate the impact of this parameter on the RPE functionality upon grafting in the RCS rat model [39]. Rat PRs remained preserved to the same extent whatever the age of the donor (from 10 to 49 years old) [39].

Autologous RPE cells are harvested from a nasal location and grafted in another site with depleted RPE. The surgical approach was first experimented in rabbits [40]. Patients with the wet form of AMD were operated on to remove a choroidal neovascularization and, on the same procedure, grafted with autologous RPE freshly harvested [41]. Eight of 13 patients showed a visual acuity improvement after 17 months. A larger cohort was then studied but limited visual improvements were reported, probably due to the poor prognosis expected from these patients [13].

PR sheets obtained from cadavers were transplanted into 8 patients with advanced RP without any signs of visual improvements (a rescue of central vision or a delay in visual loss) [42].

These different studies demonstrated the feasibility for the use of human adult RPE cells or PRs harvested from cadavers as a source for cell therapy. The major limitation for the use of allogeneic cells from cadavers and autologous RPE is the quantity of cells obtained, thus limiting the large-scale use due to a low amplification potential. As for fetal cell sources, another concern is related to the variations from one batch to another due to the heterogeneity of donors. Finally, autologous cell harvesting can generate complications due to the surgery. A robust industrialized manufacturing process based on another cell source is required to treat the millions of potential patients.

In amphibians, endogenous RPE cells are able to proliferate and reconstitute retinal cells after injuries [43]. Unfortunately, such plasticity does not exist *in vivo* for human RPE cells. The proliferation potential of adult RPE cells was recently extended with the identification of a specific subpopulation of RPE cells (2-3%) retaining self-renewing properties *in vitro* [44]. These cells named RPESCs could be harvested from cadavers (22- to 99-year-old donors) and express stem cell-like properties. Upon grafting, these cells adopt a typical RPE morphology in rabbits and preserve PRs in the RCS rat model [45, 46]. The maturation stage of RPE derived from RPESCs is an important criterion prior to transplantation in rodents, a 4-week maturation period being the most efficient for vision rescue [47].

### 3.3. Alternative Cell Sources

Ideally, the cell source should allow obtaining directly or indirectly PRs or RPE cells. However, many publications described a trophic effect engendered by the grafted cells that were not directly related to RPE cell functions. In addition, some RPE typical functions like phagocytosis, while specifically regulated, are not restricted to this cell type. That is the reason why alternative cell sources, which are not typical retinal cells, were proposed for cell therapy of the eye. It includes iris pigment epithelium (IPE), Schwann cells, fetal brain-derived neural progenitors, bone marrow mesenchymal stem cells (MSCs), retinal neurospheres, and umbilical cord stem cells.

IPE cells possess phagocytic properties and form a monolayer with tight junctions suitable to form a de novo blood-retinal barrier [48]. Human and rat IPE cells were transplanted into RCS rats through a transcleral route and were able to preserve PRs [49, 50]. Human autologous IPE obtained following local anesthesia were subcultured 1-2 months and grafted in wet AMD patients (from 49 to 85 years old) [51]. While the visual acuity was better than before the surgery, no significant improvement compared to that of the control group was reported [51, 52].

As Schwann cells secrete a variety of trophic factors necessary for PR survival, they were grafted in the RCS rat model [53]. Retinal functionality and PR survival were improved, and the same effects were observed in another RP mouse model [54]. Such cells were obtained from the rat sciatic nerve, but in humans, they could be obtained from autologous sural nerves.

Like Schwann cells, human cortical progenitors are able to secrete trophic factors that could sustain the survival of PRs [55, 56]. The human fetal material was obtained from brain tissue at 21 weeks of gestation and cultured as neurospheres for several passages [55]. These cells preserved the vision of RCS rats for 280 days [56]. When grafted in wild-type monkeys, they did not alter the retina's electrical response to light suggesting that the grafted material is safe [57]. The company StemCells Inc. generated a cell line from a single fetal brain tissue that is suitable for clinical trial [58]. When these cells were transplanted in an RCS rat, the visual phenotype was preserved [59, 60]. A clinical trial was launched with 16 patients with geographic atrophy secondary to AMD without any results published yet [61].

Bone marrow MSCs are precursors of the bone, cartilage, and adipocytes *in vivo*, and they provide a suitable environment for the hematopoietic stem cell niche [62]. MSCs could adopt an RPE-like morphology at two months postsurgery and rescue the retinal degeneration in RCS rats [63, 64]. The effect appeared to be mediated by the release of trophic factors [64].

Some rare and quiescent cells located in humans in the pars plicata and pars plana of the retinal ciliary margin can be stimulated with bFGF to proliferate *in vitro* [65]. They can be amplified as neurospheres and they are named retinal neurospheres (RNS) [66]. These cells could be isolated from early postnatal to 70-year-old postmortem donors. RNS are suggested to be able to differentiate into retinal and RPE cells upon specific culture conditions [67, 68]. However, this potential is controversial as this differentiated state could be only due to the expression of ectopic markers rather than a true differentiation [69, 70]. Future studies would clarify their potentiality and their therapeutic effect.

Human umbilical tissue-derived cells (hUTCs) are another cell source that has high amplification potentials: at least 1 × 10^17^ cells could be generated from one single donor [71]. hUTCs are derived from the human umbilical cord following normal births. When grafted into the subretinal space of RCS rats, hUTCs improved their visual phenotype [71]. This effect is also mediated by the secretion of trophic factors like the brain-derived neurotrophic factor (BDNF).

### 3.4. Pluripotent Stem Cell Sources

Human pluripotent stem cells (hPSCs) comprise human embryonic stem cells (hESCs) and human-induced pluripotent stem cells (hiPSCs). hESCs were first derived in 1998 from the inner cell mass of a human embryo at the blastocyst stage [72]. They are pluripotent which signifies that they can be differentiated in virtually any cell type of the adult body if they are provided with appropriated signalling cues. Their second characteristic is their self-renewing potential. They can be amplified for an unlimited number of passages without alterations of their fundamental properties.

hiPSCs are obtained from any differentiated cell of the human adult body after forced expression of a cocktail of pluripotency factors [73]. While not strictly identical to hESCs, they share their fundamental characteristics of pluripotency and self-renewal [74]. hiPSCs or hESCs differentiated into RPE cells are functional *in vivo* and restore, to the same extent, visual functions of rodents with dystrophic retinas [75, 76].

All the previously cited cell sources for retinal cell therapy have a lot of limitations in terms of supply chain, donor to donor variability, and scale up (limited cell amplification). Moreover, all strategies based on nonretinal cells may only have a transient effect and will not replace lost PRs or RPE cells. With the emergence of hPSCs, the potential to generate every cell type of the human retina *in vitro* has redirected all the efforts to the development of robust differentiation protocols from these cells, which could be used for disease modelling or cell therapy [77].

## 4. Differentiation Strategies of hPSCs

The differentiation of hPSCs into the various retinal cell types follows the sequential steps of the normal development occurring in the human embryo as identified by time-dependent expression of marker characteristics of these steps [78]. While spontaneous differentiation generates poor yield of cells of interest, a number of protocols take advantage of the knowledge acquired regarding mouse and human embryonic developments to direct the differentiation and increase the rate of material suitable for transplantation.

### 4.1. Production of RPE Cells

hPSCs grow as small colonies in a medium containing the fibroblast growth factor (FGF2) that maintains their undifferentiated state. The withdrawal of FGF2 from the culture medium is sufficient to initiate the differentiation. After few weeks of culture under these conditions, pigmented patches start to appear in the culture dish. These patches could be isolated and further amplified to obtain RPE cells [79–84]. In other approaches, hPSCs could be dissociated and cultured as a cell suspension without FGF2. hPSCs then form cell aggregates, which are called embryoid bodies [85, 86]. Embryoid bodies are then cultured in adherence, and when pigmented patches appear, they are further manually collected and amplified [87]. The amplification potential of the RPE cells generated is limited, and the cells may undergo an epithelial-to-mesenchymal transition after four to six passages [88, 89]. Such amplification potential could be expanded through Rho-associated, coiled-coil protein kinase (ROCK) inhibition using small molecules [89].

The efficacy of RPE differentiation could be improved by the addition of growth factors or small molecules in the culture medium [90]. To trigger neuroectoderm induction, inhibitions of TGF*β* and/or BMP pathways are required [91]. Small molecules like SB431542 (TGF*β* inhibitor) could be added in the culture medium [75, 92]. The WNT pathway inhibition may also be used to induce an anterior neural fate [93, 94]. Other factors known to induce an RPE fate could also be applied [95, 96]; some of them like chetomin were identified through high throughput screening [97]. An activation of the WNT pathway later during the differentiation could also increase the yield of RPE cells [98].

To further progress in the large-scale production of RPE cells, automated systems of manufacturing could help increase the reproducibility and consistency from batch to batch of the therapeutic cells [99].

### 4.2. Production of the Neural Retina

**Figure 2:**
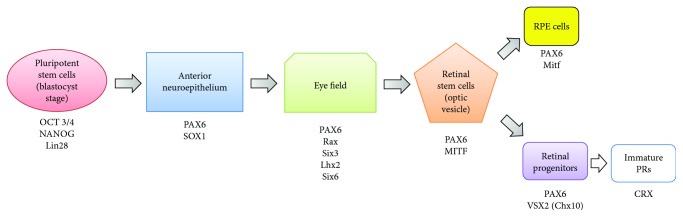
Scheme describing the sequential developmental steps to generate RPE cells and PRs from hPSCs and the different markers that could be used to discriminate between them.

The differentiation of hPSCs into the neural retina follows the same embryonic developmental pathway [78]. The anterior neural plate is regionalized and becomes the eye field, which is characterized by a subset of eye field transcription factors (EFTFs) [100]. EFTFs are highly conserved among species, and their expressions allow confirmation of the stage of development (Figure 2). Then an evagination leads to the formation of the optic vesicle which contains retinal stem cells that will give rise to all cell types (RPE and neural retina). A patterning of retinal stem cells distinguishes then retinal progenitors from immature RPE cells which correspond to the first differentiated cell type that appears [100]. PRs arise from the retinal progenitors sequentially during development with partial overlap with the other retinal subtypes [101].

A variety of protocols have been developed to generate PRs, mainly through the formation of organoids that resemble the optic vesicle or optic cup stage *in vitro* and that contain the neural retina and RPE cells [83, 84, 102–107]. As for RPE production, a variety of factors could be applied to the culture media in time- and dose-dependent manners to increase the proportion of the cell type of interest. Some degree of retinal lamination similar to *in vivo* development could be achieved, but culture times are usually long to obtain mature PRs. Some protocols obtain PRs mixed with other cell types through a 2D differentiation [108, 109].

Due to the heterogeneity of the cell types obtained, different strategies were developed to obtain transplantable PRs that are purified and depleted from mitotic cells. The selection and isolation, using a panel of cell surface markers like CD73 specific to PRs in the retina or through a negative selection with markers not expressed in PRs, were successfully achieved [110–114]. To date, though, few protocols to generate PRs are compatible with a clinical use [84, 106].

## 5. Formulation and Delivery of the Cell Therapy Product

**Figure 3:**
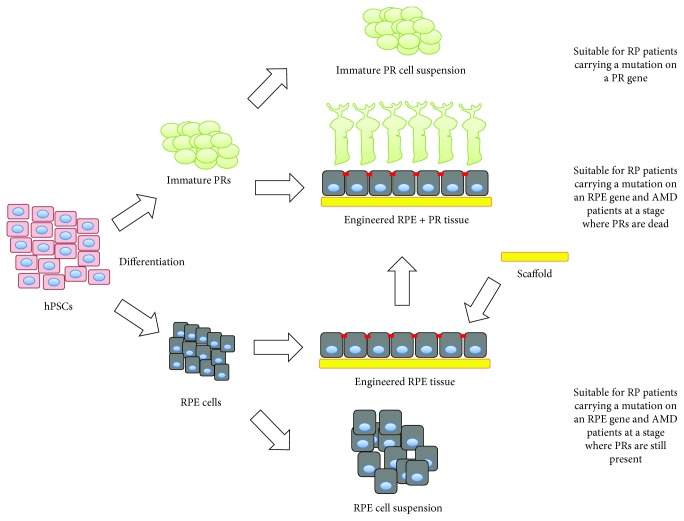
Scheme recapitulating the strategy to formulate a cell or a tissue therapy to treat patients with various stages of retinal degeneration.

To be successful, the cell therapy should lead to the replacement of the dead or defective cell type. The way to formulate it, for delivery into the recipient eye, has major consequences on therapeutic outcomes in patients. The scheme below recapitulates the different modes of delivery for a potential cell or tissue replacement therapy (Figure 3).

### 5.1. RPE Cell Formulation

When RPE cells derived from hPSCs are manufactured, they could be banked and cryopreserved in liquid nitrogen at the end of the process. It allows determining the quality of the production in terms of purity, safety, potency, and stability of cells over time of cryoconservation. From such banks, different formulation strategies were proposed for the delivery to patients: either as cell suspension or after a prior step of epithelial reconstitution *in vitro*. Cell suspension formulation engenders a simplified logistic. Indeed, the RPE cell cryovial could be sent directly to the hospital without additional culture steps. On site, the cells are thawed, washed from cryoprotecting medium, and formulated as a suspension in a syringe [86, 115]. First clinical trials for dry AMD and Stargardt's disease were conducted using this strategy [86, 115, 116]. As first-in-man, the primary concern was the safety of the cell therapy but some improvements in visual acuity were reported despite initial poor vision of patient cohorts. These effects were sustained for 4 years in some patients [117].

However, such cell suspension formulation raises some concerns about efficient integration, functionality, and survival of the grafted material. RPE cell functions rely on its epithelial organization with the secretion of cytokines, the resistance to a stressed environment, or the apicobasal polarization of key proteins [45, 118–120]. When implanted in immunocompromised rats, the survival of RPE cells injected as a suspension was reduced compared to cells injected as an epithelial tissue [121]. When Bruch's membrane, in which RPE cells adhere to form the epithelium, is compromised as it could be in AMD, cells injected as a suspension will not integrate properly [122]. Regarding functionality, the idea is that the transplantation of an already formed epithelium should be effective more rapidly compared to a cell suspension, which would require forming an epithelium in a diseased retina. Our group had demonstrated this paradigm by grafting the same cells as a suspension or as an epithelium using a biological scaffold in the RCS rat acute model of retinal degeneration [79, 123]. The electrical response of the retina to light and the preservation of PRs were significantly improved and for a longer period of time when the cells were grafted as a tissue composed of an RPE monolayer on top of a biological scaffold [79].

Several strategies were developed to generate an RPE epithelium ready for transplantation including the use of a supporting scaffold. Such a scaffold would also replace Bruch's membrane. The selection of a candidate scaffold should meet the following properties: mechanical resistance and flexibility for easy handling, permeability to allow exchange of nutrients and waste materials, thickness that is compatible with a subretinal implantation, and, if biodegradable, nontoxic byproducts [3, 119, 124, 125].

Nonbiodegradable synthetic polymers like the ultrathin parylene or the porous polyester are currently tested as scaffolds for the RPE tissue preparation in ongoing clinical trials [20, 121, 126–129]. Poly-lactic-co-glycolic acid or PLGA, a biodegradable polymer, supports the culture of RPE cells and will be also tested in a clinical trial [130, 131]. These synthetic polymers are easy to produce and thus could be used to prepare treatment doses for the millions of patients [45, 121, 132]. The potential toxicity of these polymers formulated as a scaffold should be carefully evaluated when implanted into the eye of animal models. While being approved for some clinical applications (including eye implants), the PLGA under a specific formulation induced a severe immune reaction into the eye of nonhuman primates and rabbits [133]. Biological scaffolds are also under investigations like Descemet's membranes or human amniotic membranes [79, 134, 135]. This type of support is more biologically relevant and mimics at some points the endogenous Bruch's membrane. The human amniotic membranes are classically prepared, cryopreserved as small patches, and used for clinical applications in many countries. Such banks render the human amniotic membranes attractive as a biological support for the RPE tissue replacement. A clinical trial with this support is planned to start in 2019 [3, 79]. A Japanese group developed an ingenious alternative to the use of scaffolds [75, 136]. This group used a collagen matrix to support the epithelial formation. Once formed, the epithelium is detached from the support using a collagenase treatment. So far, the results of one patient with a wet form of AMD have been published using this strategy, based on autologous hiPSC-RPE transplantation [136]. The visual acuity of this patient was maintained at one year. However, the five other planned patients were not treated and the clinical trial was suspended [136, 137]. The genetic defects found in the cells prepared from the second patient in addition to a change of strategy toward the use of allogeneic hiPSC-RPE are at the origin of this clinical trial's stoppage.

Despite being physiologically relevant, the tissue formulation complicates drastically the logistical procedures. First, it requires a prior culture step of RPE cells to reconstruct an epithelium which leads to mobilization of a manufacturing suite for each patient. The scheduling of the surgery should be anticipated to generate the grafting material on time. Such tissue therapy requires the development of specific injection systems in order to correctly graft the polarized epithelial tissue [79, 128, 138]. All these new constraints increase the costs. However, the field is moving in this direction and several clinical trials were initiated with already promising first results [128, 129, 136]. In a preliminary report, one of 2 patients with severe exudative AMD who was not able to read before the implantation was capable to do so at one year postsurgery [128]. The graft in this study was composed of a hESC-RPE monolayer, obtained from a spontaneous differentiation method and seeded on a coated polyester scaffold. Any conclusion with the functional benefits of this treatment should be taken carefully as for an early safety clinical trial, there is no control group and the number of patients is extremely limited. Moreover, three serious adverse events were reported (two of them needing a surgical intervention). All these adverse events were successfully treated and were suggested not to be related to the grafted cells [128]. Another Japanese clinical trial which uses allogeneic hiPSC-RPE cell suspension reported also a serious adverse event needing a surgical intervention. So far, the origin of this event seems unrelated to the grafted cell material (https://www.japantimes.co.jp/news/2018/01/17/national/science-health/first-serious-reaction-ips-derived-retinal-cell-transplant-reported-kobe/#.WxFnl1OUtTa).

### 5.2. PR Cell Formulation

The stage of development of grafted PRs is crucial for their correct integration [139–141]. Indeed, mature PRs will be less prone to establish the short synaptic connection required for their functionality. Studies conducted in mice demonstrated that early postnatal retinas would be the best source material for transplantation due to their ability to integrate in the preexisting circuitry [140, 142]. This stage of retinal development, corresponding in mice to the time window of the rod PR production, was thoroughly characterized in order to define the markers that would help to identify similar stages during human pluripotent stem cell differentiation into PRs [111, 113, 143].

Different attempts were described in the literature where PRs were dissociated and injected as a cell suspension [106, 114, 140, 142, 144]. First interpretations concluded that such cells are able to integrate in the preexisting circuitry and are able to elicit some kind of visual recovery. In the recent years, a couple of studies came to the conclusion that such integration is a rare event and most of the cells remain in the subretinal space [145–147]. The previous misinterpretation came from an unexpected phenomenon of cytoplasmic transfer between donor cells and endogenous remaining photoreceptors. By such exchange of materials, fluorescent proteins used to trace the donor's cells were found in endogenous photoreceptors. The exchange did not involve the transfer of the nucleus which means that the protein transfer needs to be sustained to achieve function [145–147].

Functional outcomes of PR transplantation remained limited due to the few numbers of PRs integrated or which were involved in the cytoplasmic exchange [142, 145–147]. Indeed, visual parameters evaluated by electrical responses of the retina to light or behavioural paradigms (like optokinetic reflexes or pupil light responses) in rodent with degenerated retinas were moderately or not improved [114, 142, 144, 148]. The cell suspension delivery could explain this low integration [142, 149]. A study described however an improved electrical response to light in the retina following transplantation of PRs in mice with retinal degeneration, which could be a result of a cytoplasmic material transfer [150].

Few studies were published using PR sheets. Retinal organoids derived from pluripotent stem cells were cut into 0.5 mm sections prior transplantation [151–154]. In that context, PRs formed synaptic connections and developed inner and outer segments. However, visual recovery was not achieved using classical methods of evaluation in immunocompromised mice with retinal degeneration, probably due to the low number of the grafted material [151, 153]. Moreover, the PR sheets tend to form rosettes, and outer segments were separated from the support of endogenous RPE cells inside these rosettes [151, 153, 154]. Such organization could impact on the survival and functionality of the grafted material, and further development is required to avoid such structures.

## 6. Future Directions

Our understanding of the mechanisms of the hPSC differentiation into RPE cells and PRs has considerably progressed. However, we still need to optimize the way to formulate the tissue to be grafted in order to achieve the best functional recovery. This is particularly true to produce a complex retinal tissue containing layers of PRs and RPE cells suitable for grafting. The proof of concept of visual recovery following transplantation in models of retinal degeneration is globally still missing, as only some forms of rescue were observed using tests that require low vision performances. This will be achieved only by a future optimization of the grafted material in order to be able to graft PRs and PR/RPE sheets that would not fold or form rosettes in the recipient eye. A microstructured 3D scaffold that would guide the correct orientation of PRs is a promising strategy to generate organized layers of PRs [155].

The field is progressing carefully, and first clinical trial results are starting to be published [86, 115, 128, 129, 136]. The RPE cell therapy is now tested by many laboratories using various formulation strategies, while the PR cell therapy is still at the preclinical stage (Table 1). The time taken to develop these hPSC-based treatments is necessary to ensure patient's safety. The recent publication showing the danger of “stem-cell” clinics in the United States is here to remind us that, without rigorous methodology and robust preclinical data, patient's vision could be irremediably damaged [156].

Most of ongoing and planned clinical trials use allogeneic material and therefore are using an immunosuppression therapy. The immunosuppression strategy is intensely debated despite the fact that the eye is generally considered as an immune-privileged site [45, 157–160]. Recently, the transplantation of GFP-labelled allogeneic iPSC-RPE cells in a pig model resulted in an innate immune reaction, despite the survival of the grafted cells until the end of the study (3 weeks) [161]. Current strategies are ranging from local immunosuppression to high systemic immunosuppression [86, 115, 128, 129]. A high immunosuppression engenders many side effects. If this treatment is demonstrated to be mandatory, the use of hPSC banks with known human leukocyte antigen (HLA) genotypes that could match with one of the recipient retinas will be an attractive alternative to reduce the risk of graft rejection [75, 162–164]. As an example, it was calculated that 10 selected homozygous HLA-typed donors could match 37.7% of the UK population [165]. To cover 93% of the UK population, 150 selected donors are required. Thus, a limited number of hPSC lines are needed to limit the use of heavy immunosuppression treatments. A clinical trial was recently launched in Japan with this HLA-matched approach, and a patient with a wet form of AMD was grafted with RPE cell suspensions (http://www.cdb.riken.jp/en/news/2017/topics/0404_10343.html).The results of this clinical trial will confirm if this strategy is sufficient to prevent the graft rejection.

## 7. Conclusion

Cell therapy for retinal dystrophies is at the forefront of the clinical investigation compared to other diseases in which such treatment approach could be applied. Indeed, with the ease to access and the available imaging techniques as well as the sophisticated functional assays, retinal diseases serve as a proof of concept of regenerative medicine applications. However, we should remain cautious with the completion of all milestones before treating patients. In particular, the quality, safety, and expected functionality of the cell therapy product to be delivered to patients should be precisely evaluated. In the coming years, results from many clinical trials will help determine the best formulation strategy to be applied for each category of patients as well as the level of immune suppression required.

## Figures and Tables

**Table 1:** Current or planned clinical trials based on hPSCs to treat RP, Stargardt's macular dystrophy (SMD), or AMD.

Stage of development	Targeted disease	Sponsor/company	Therapy
Phases I and II NCT02563782, NCT01469832, NCT01344993, NCT01345006	AMD, SMD	Astellas Institute for Regenerative Medicine	Allogenic hESC-RPE, cell suspension injection
Phase I/II NCT01674829	Dry AMD	CHA Biotech Co. Ltd.	Allogeneic hESC-RPE, cell suspension injection
Phase I/II NCT02286089	Dry AMD, GA	Cell Cure Neurosciences Ltd.	Allogeneic hESC-RPE, cell suspension injection
Phase I/II NCT02590692	Dry AMD, GA	Regenerative Patch Technologies LLC	Allogeneic hESC-RPE, epithelium on a parylene membrane
Phase I/II	AMD	RIKEN Centre for Developmental Biology	Autologous and allogenic hiPSC-RPE, epithelium without substrate
Phase I NCT01691261	Wet AMD	Pfizer/London Project to Cure Blindness	Allogenic hESC-RPE, epithelium on polyester membrane
cGMP optimization	AMD, SMD, RP	Cellular Dynamics International/NEI	Autologous hiPSC-RPE, epithelium on biodegradable scaffold
Phase I/II	RP with genetic defects in LRAT, MERTK, RPE65	I-Stem/Institut de la Vision	Allogeneic hESC-RPE, epithelium on amniotic membrane
Preclinical	AMD, best disease, LCA	HMC, Israel	Allogeneic hESC-RPE, cell suspension injection
Phase I/II NCT03046407, NCT02749734, NCT02755428	Dry AMD, SMD	Chinese Academy of Sciences, Southwest Hospital, China	Allogeneic hESC-RPE
Phase I/II NCT02903576	Dry AMD, wet AMD, SMD	Federal University of São Paulo	Allogeneic hESC-RPE as cell suspension versus as a sheet on a polymeric substrate
